# Natural exposure to Chikungunya virus in golden-headed lion tamarin (*Leontopithecus chrysomelas*, Kuhl, 1820) from non-protected areas in southern Bahia, Brazil: Implications and significance

**DOI:** 10.1371/journal.pntd.0012695

**Published:** 2025-01-24

**Authors:** Sofía Bernal-Valle, María Angélica Monteiro de Mello Mares-Guia, Filipe Vieira Santos de Abreu, Fabrício Souza Campos, Cirilo Henrique de Oliveira, Antônio Victor Veloso Ramos, Reizane Pereira Lordelo, Kristel De Vleeschouwer, Leonardo de Carvalho Oliveira, Hllytchaikra Ferraz Fehlberg, Ana Maria Bispo Filippis, Bergmann Morais Ribeiro, Paulo Michel Roehe, Anaiá da Paixão Sevá, Danilo Simonini-Teixeira, George Rego Albuquerque

**Affiliations:** 1 Department of Agricultural and Environmental Sciences, Universidade Estadual de Santa Cruz, Ilhéus, Bahia, Brazil; 2 Núcleo de Atendimento e Pesquisa de Animais Silvestres (NAPAS), Universidade Estadual de Santa Cruz, Ilhéus, Bahia, Brazil; 3 CENIBiot Laboratory, The National Center of High Technology (CeNAT-CONARE), San José, Costa Rica; 4 Laboratory of arboviroses and hemorrhagic viruses, Instituto Oswaldo Cruz, Fiocruz, Rio de Janeiro, Rio de Janeiro, Brazil; 5 Insect Behavior Laboratory (LACOI), Instituto Federal do Norte de Minas Gerais, Salinas, Minas Gerais, Brazil; 6 Institute of Basic Health Sciences, Universidade Federal do Rio Grande do Sul, Porto Alegre, Rio Grande do Sul, Brazil; 7 Projeto BioBrasil, Antwerp Zoo Centre for Research and Conservation, Antwerp, Belgium; 8 Programa de Pós-Graduação em Ecologia e Conservação da Biodiversidade—PPGECB, Universidade Estadual de Santa Cruz, Ilhéus, Bahia, Brazil; 9 Bicho do Mato Instituto de Pesquisa, Belo Horizonte, Minas Gerais, Brazil; 10 Baculovirus Laboratory, Department of Cell Biology, Institute of Biological Sciences, University of Brasilia, Brasília, Federal District, Brazil; McGill University Faculty of Medicine and Health Sciences, CANADA

## Abstract

Chikungunya virus (CHIKV) is primarily associated with non-human-primates (NHPs) in Africa, which also infect humans. Since its introduction to Brazil in 2014, CHIKV has predominantly thrived in urban cycles, involving *Aedes aegypti* mosquitoes. Limited knowledge exists regarding CHIKV occurrence and implications in rural and sylvatic cycles where neotropical NHPs are potential hosts, from which we highlight *Leontopithecus chrysomelas* (Kuhl, 1820), the golden-headed lion tamarin (GHLT), an endangered species endemic to the Atlantic Forest (AF) in Southern Bahia State, Brazil. The present study investigated wild GHLT groups across two municipalities, Ilhéus and Una, Bahia. Surveys were conducted in three groups within cocoa agroforests (*cabrucas*) in Ilhéus, and four groups in anthropized forest and agroforestry fragments in Una, between 2021 and 2022. Thirty-two GHLT specimens were captured and chemically immobilized, examined and submitted to blood sample collection; nine specimens were later recaptured in 2022, totaling 41 samples. CHIKV viremia was not detected in any specimens (as assayed by RT-qPCR). Plaque reduction neutralization test (PRNT_90_) detected CHIKV antibodies in two (6.3%) GHLTs, with 10–20 antibody titers. Seroprevalence in 2021 was 5.6% and in 2022 was 8.7% with an incidence of 4.5%, whereas, a male adult tested seropositive in both years, suggesting either natural re-exposure and antibody maintenance over time. All samples tested seronegative for Mayaro Virus. Eight mosquito species from the Culicidae family were collected, identified and assayed for CHIKV genomes, showing negative results. This study provides the first evidence of natural CHIKV exposure among free-living GHLTs in Brazil, emphasizing their susceptibility and potential role as reservoirs. These findings underscore the possible consequences of anthropic disturbances in the Brazilian AF, without a seroprevalence difference between non-protected forest formations, agroforest fragments and various mosaic farming landscapes in South Bahia, and highlight the importance of conservation efforts for this endemic and endangered primate species.

## Author summary

Chikungunya virus (CHIKV) is an arbovirus, where in Africa non-human-primates (NHPs) are hosts of the sylvatic transmission cycle. Since 2014 in Brazil CHIKV is maintained in urban cycles, involving humans and *Aedes aegypti* mosquitoes. We have limited knowledge regarding CHIKV occurrence and implications in rural and sylvatic cycles where neotropical NHPs are present. In 2021 and 2022, we studied Culicidae mosquitoes and *Leontopithecus chrysomelas* (golden-headed lion tamarin -GHLT-), an endangered species endemic to the Atlantic Forest in Southern Bahia State, Brazil, at Ilhéus and Una municipalities. There was no evidence of genetic material of the virus in the GHLT and mosquitos, but we did find neutralizing antibodies as evidence of natural CHIKV exposure among free GHLT, that were present for at least five months. This could mean that GHLTs i) are susceptible to CHIKV infection, ii) may have a potential role as reservoir, and iii) are also important to include it in the epidemiological surveillance of arboviruses. Although we did not find a difference in the antibody titles in the tamarins between inhabiting the different land covers and soil uses, this endangered species could be infected in anthropogenic altered regions such as rural areas, as a consequence of anthropogenic pressure.

## Introduction

Chikungunya virus (CHIKV) is an alphavirus and zoonotic arbovirus, belonging to the *Togaviridae* family. It was initially reported in forested areas in Africa, where wild vector mosquitoes of the genus *Aedes* and non-human-primates (NHPs) hosts are essential to the cycle maintenance [1–3]. This virus was historically confined to the Old World [4,5], however, since 2004, it has undergone significant geographic expansion [6]. The CHIKV Asian lineage was introduced to regions of Latin America in 2013, with subsequent detection in the Amapá State, Northern Brazil, in 2014. Simultaneously, the African lineage (East/Central/South African, ECSA) was detected in Bahia State, Northeastern Brazil, also in 2014. Following autochthonous transmission, the virus rapidly spread to other states across the country [7–10]. CHIKV belongs to “Semliki Forest” Complex (SFV), along with Mayaro Virus (MAYV) that has a more restricted distribution pattern in neotropical regions [11,12]. Notably, in the American Continent, these two viruses are able to co-circulate [13].

Nowadays, in the Brazilian epidemiological scenery, CHIKV, alongside other arbovirus such as Dengue (DENV) and Zika (ZIKV) (*Orthoflavivirus*, Flaviviridae) are major agents of arboviruses-associated diseases in urban areas transmitted to humans by *Aedes aegypti* mosquitoes [8]. The main symptoms of these arboviruses are acute fever with severe and chronic arthralgia in humans, and consequently, these viruses represent an important public health concern, as they can also co-circulate and co-infect their hosts, showcasing remarkable adaptability to diverse environments [12]. Unlike the African continent, where the CHIKV depends on enzootic amplification in NHPs [1,2], and can lead to clinical sings that mirrors the response observed in humans [14], in the Americas, this virus seems to have deviated from this dependence pattern and efficiently sustains an urban transmission cycle involving human-mosquito-human dynamics, with *Ae*. *aegypti* serving as the primary vector [6,15,16] and along with *Ae*. *albopictus*. These two mosquito species that occur mostly in anthropized areas, are widespread in Brazil [7].

Our understanding of mosquito diversity in forested areas adjacent to or within urban and rural regions of Brazil remains limited, as highlighted in other studies [9,17–19]. However, a few investigations conducted in an urban park in Bahia (BA) State, situated within the Atlantic Forest (AF) [9] as well as in its municipalities of Una and Ilhéus [18], have identified mosquito species with potential as pathogen vectors. Specifically, sylvatic species such as *Haemagogus leucocelaenus* and *Aedes terrens* as well as the periurban/rural species -*Ae*. *albopictus* -, which are found in Brazil, have demonstrated vector competence for CHIKV under laboratory conditions, similar to *Ae*. *aegypti* [20,21]. This suggests a potential risk for the establishment of a sylvatic cycle of CHIKV in neotropical regions with NHPs presence, as these vectors can amplify the virus sufficiently to infect wild populations [20].

Several factors contribute to the transmission cycles of CHIKV and other arbovirus, including environmental variables, climate change, modified land like deforestation and urbanization, and other socioeconomic factors. Studies have shown that inadequate sanitation and water supply significantly influence the incidence of arboviruses in Colombia. Additionally, barriers to health services and the influx of populations due to political instability have exacerbated the transmission of dengue, ZIKV, and CHIKV in these regions [22]. Climatic factors such as temperature and rainfall also play a crucial role, with ZIKV and CHIKV outbreaks in Rio de Janeiro linked to major rainfall events with a lead time of about three weeks [23]. These factors increase interactions between hosts, vectors, and viruses, facilitating the emergence and re-emergence of infectious agents. Understanding the role of other vertebrate hosts, especially NHPs, is essential for the epidemiological surveillance of arboviruses [1,24,25].

Despite experimental researches in Old World NHPs demonstrating clinical signs and development of neutralizing antibodies against CHIKV [14], as far as the authors know, there is no information in New World NHPs. Based on natural exposure to CHIKV in areas with intense local transmission in humans, the Neotropical NHPs from urban or peri-urban areas, such as *Sapajus* spp., and *Callithrix jacchus*, from Northeastern Brazil may not sustain outbreaks of the virus as they exhibit lower susceptibility to CHIKV infection, yielding low antibody titles [25]. However, there is a lack of information regarding the infection of CHIKV and the health and conservation consequences of this virus in free-ranging golden-headed lion tamarin (GHLT), *Leontopithecus chrysomelas* (Kuhl, 1820) (Callithrichidae) [25,26], an endangered primate species currently limited to the AF in Southern Bahia State[27,28]. Unfortunately, mainly because of anthropogenic pressure, its population has seen a decline of approximately 60% over the past few decades, coupled with a 42% reduction in its geographic range [29]. GHLTs are predominantly found in agricultural mosaic land uses, forest fragments and mostly cocoa agroforests, known as *cabrucas* (agroforestal system with cocoa trees below the forest tree canopy), in the eastern portion of their geographic range in AF [30], including Ilhéus and Una municipalities.

Arboviruses hold a crucial hole not only in public health, but also for animal health in the context of conservation efforts within the One Health framework. Some arboviruses can indeed cause illness or death in New World NHPs, such as Yellow Fever virus (YFV) in natural exposure [31–33] and ZIKV, mainly in experimental conditions [34]. However, our understanding of the interactions and disease dynamics, particularly including wild animals like NHPs [24] remains limited. This gap in in knowledge highlights the importance of strengthening epidemiological monitoring and research efforts across diverse geographical regions and primate species. To unravel these complex systems, it is imperative to strengthen epidemiological monitoring strategies, emphasizing sustained, long-term surveillance efforts. Such initiatives empower researchers to delve into the intricate interplay and characteristics of each component—animal, human, and their environment—thereby deciphering the dynamics of diseases. This knowledge, in turn, informs epidemiological surveillance and vector control programs.

In an effort to elucidate the occurrence of alphavirus infections in *L*. *chrysomelas* (Kuhl, 1820), from South Bahia, we hypothesize that GHLTs inhabiting different scenarios of anthropogenically disturbed forest fragments of AF, such as *cabrucas* and secondary forests with other agroforests and farming land uses within rural and periurban landscapes, may play a role as hosts to CHIKV.

## Methods

### Ethics statement

The research adhered strictly to the ethical guidelines established by the American Society of Primatologists for the treatment of non-human-primates (NHP) and complied with relevant laws governing research conducted in Brazil. Approval for the study was obtained from the Ethics Committee for the Use of Animals in Research of the Universidade Estadual de Santa Cruz (CEUA/UESC, Approval No. 023/20). Additionally, authorization was granted by the Institute Chico Mendes de Conservação da Biodiversidade (ICMBio), a division of the Brazilian Environmental Agency (ICMBio–SISBIO, Permit No. 75734–1), and the National System for the Management of Genetic Heritage and Associated Traditional Knowledge (SISGEN, Registration No. AF40BCA).

### Study area and animals

The study was conducted between June 2021 and May 2022, focusing on wild populations of GHLTs in the eastern region of their distribution. The area is situated in south Bahia State, within the tropical AF- biome -. With an Af climate type, according to the Köppen´s classification, characterized by a hot and humid climate without a well-defined dry season [35], with a mean temperature of 24°C and an annual rainfall ranging 1200 to 1800 mm, evenly distributed throughout the year [36,37].

**Fig 1:**
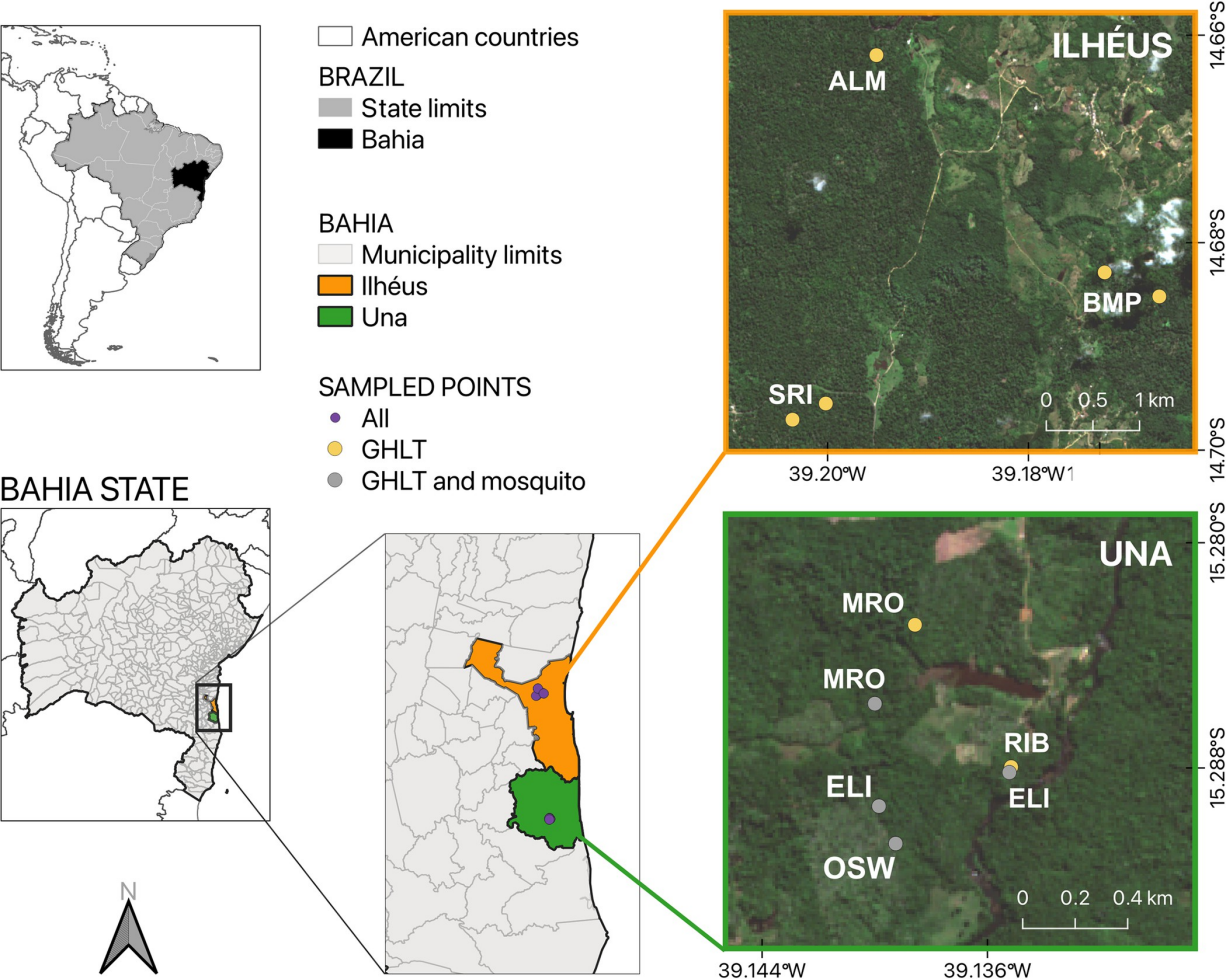
Sampling areas of the groups of free-living *Leontopithecus chrysomelas*, Kuhl, 1820 (Golden-Headed Lion Tamarin, GHLT) and mosquitoes in Una and Ilhéus municipality, south Bahia State, Brazil, collected during the years 2021 and 2022. Group name: Almada (ALM), Bom Pastor (BMP)–sampled in two points, Santa Rita (SRI)–sampled in two points, Ribeiro (RIB), Elias (ELI)–sampled in two points, Manoel Rosa (MRO), and Ozawa (OZW). This image of basemap was performed by using satellite images of bands green, red and infrared (10m resolution) of Sentinel-2 (date: 22/06/09), which are of public domain available from *U*.*S*. *Geological Survey* at Copernicus browser (https://browser.dataspace.copernicus.eu). Map generated with the free and open source geographic information system (QGIS,version 3.32; licensed under general public license [40]).

Two different scenarios were defined to identify the infection of CHIKV in GHLTs. Three groups were located in the Ilhéus municipality, examined in predominantly private *cabrucas* landscapes, corresponding to Almada (ALM), Bom Pastor (BMP), and Santa Rita (SRI) farms [38]. The farm names also correspond to the GHLT family groups names, indicating that these species live in small family groups within these specific farms. Additionally, four groups were within the Una municipality, with a mixed anthropized forest area, featuring secondary forest patches, various agricultural plantations (rubber, *cupuaçu*, soursop, pepper, banana, cacao, and ephemeral crops), as well as open fields and *cabruca*, located on several properties on and around the Santo Antônio farm [39]. These four groups are named Ribeiro (RIB), Elias (ELI), Manoel Rosa (MRO), and Ozawa (OZW) (S1 Table) (Fig 1). The groups BMP, SRI and ELI were sampled in two different points (Fig 1).

In the municipality of Una, mosquito capture took place in the same area as the GHLT capture points (Fig 1). However, in Ilhéus, the mosquito sampling points did not align the GHLT capture sites (S1 Table).

### Non-human-primate capture, handling and processing

The first capture period in both areas was carried out in June, July, October, and November of 2021, and the second one in March, April, and May of 2022. Banana-baited automatic “Tomahawk traps” (15.2cm x 15.2cm x 48.3cm), were strategically placed on 1.5 m high platforms, within the home range of each group of GHLTs, previously defined [41–43]. Captures were performed during day time, from 06:00h thru 17:00h. The individuals that were managed after 16:00 were released the day after, before 06:00h. After capture, the tamarins were taken to a field laboratory near the capture site, equipped with all necessary materials and sheltered from direct sunlight and rain. Following a minimum of three hours of fasting pos-capture, each individual underwent physical and chemical restraint [42,43]. Isoflurane, administered using the VetBag machine, an open system with universal vaporizer, was utilized for inhalation anesthesia [43]. To mitigate stress during anesthesia, animals were gently pressed against the back of the *Tomahawk* trap using a wood press, as outlined by Catenacci et al. (2022). Isoflurane was introduced through the Tomahawk openings to the animal’s nostril with the machine´s hose [43].

During chemical restraint for the blood samples and data collection, a comprehensive physical examination was conducted, to evaluate body condition, hydration, color of mucous membranes, size and consistency of peripheral lymph nodes, integrity of skin and coat, presence of lesions and ectoparasites or other signs of disease. Also, it included the monitoring of vital signs such as heart and respiratory rate, as well as body temperature, at every 5 minutes [43]. Additionally, morphometric, sex and stage of development data was collected. For this last characteristic, animals were classified as infant (<3 months), juvenile (3–12 months), subadult (12–18 months), and adult (>18 months) [44]. Once these animals are included in long-term monitoring projects, their individual ages are good estimated and classified, as formerly used in the project. Due to that the names of groups were previously defined and the number of troop members was known. To ensure individual identification, a 12x2.1 mm subcutaneous microchip (Transponder Partners, model PA140), was implanted in the interscapular region. Upon completion of the handling procedure, animals that had fully recovered from anesthesia were released at the same capture site, following the protocol outlined by Catenacci and collaborators (2022).

All personnel involved adhered to biosafety protocols and wore appropriate protective equipment (PPE) such as latex gloves, masks, aprons, and head dresses [42,43]. All procedures were performed by veterinarians, biologists and trained field assistants.

### Biological samples collection

Approximately 3ml of blood (not exceeding 1% of live weight) were collected from each specimen from the internal femoral vein [45,46], using a 23 x 1” gauge needle after skin disinfection. A 0.5 ml aliquot of blood was homogenized in a cryotube containing ethylenediamine tetra-acetic acid solution (1.2mg EDTA-K2/ml of blood). Serum was obtained from another 1 ml aliquot of blood, transferred to a tube with a clot accelerator (Vacuette). After clot retraction, the sample was centrifuged (DAIKI, model 80-2B-DM) for five minutes at 5000 rpm to isolate the serum [47]. Both samples were stored in cryotubes in liquid nitrogen in the field and later preserved at -80°C in the laboratory for further analysis.

### Collection and identification of mosquito vectors

Adult mosquitoes were systematically collected over a 12-day period, coinciding with the days of the NHPs captures and sampling. Mosquito collection occurred during daylight hours (09:00h – 18:00h) by a team of three individuals, dedicating up to one hour each day, resulting in a total collection time of 36 hours. Adult mosquitoes were gathered using the protected human attraction technique (APHP), following the protocols of the Secretaria de Vigilância Epidemiológica do Ministério da Saúde do Brasil [48]. This technique involved the use of oral aspirators and hand nets for collection [49]. Subsequently, mosquitoes were cryopreserved in liquid nitrogen (-196°C), transported to the laboratory, and stored at -80°C until further processing. Taxonomic identification was performed on a cold table at -20°C using a stereoscopic microscope [49] and dichotomous keys [50–52].

### Laboratory analyses

#### RT_qPCR assay for the molecular detection of CHIKV

Extraction of RNA and purification from whole blood in EDTA was performed using the EXTRACTA Kit Fast–Viral DNA and RNA (MVXA-P016 FAST), following the manufacturer’s instructions, and using 150 μl of each sample. For the extraction and purification of RNA from identified mosquitoes, pools of up to 10 individuals of non-blood-fed individuals of the same species and collection points were used. Each pool was placed in 500 μL of L-15 culture medium with 20% fetal bovine serum and homogenized using a Loccus L-Beader 24 tissue homogenizer in tubes with microbeads [53]. Following centrifugation (9600× g, 5 min, at 4°C), RNA was extracted from 140 μL of supernatant using the Qiagen RNA Viral Kit following the manufacturer’s recommendations [53].

The RT_qPCR assay for the molecular detection of CHIKV was performed according to Lanciotti et al. (2007) [54], with the GoTaqProbe 1-Step RT-qPCR System kit (PROMEGA), following the manufacturer’s instructions. In brief, 12.5μl of the master mix was combined with 0.5 μl of the enzyme (GoScript), 1.075 μl of the primer/probe mix (S2 Table), and 5.925 μl nuclease free H_2_O, and 5 μl of RNA.

The optimized run conditions were as follows: reverse transcription at 45°C for 30 min, denaturation at 95°C for 2 min, and 50 cycles of 95°C for 15 s and 60°C for 1 min [54]. Amplification was performed on a 7500 Fast instrument (Applied Biosystems), software version 2.3.

#### Plaque reduction neutralization test (PRNT_90_) for Chikungunya virus and Mayaro Virus

Initially, complement system proteins in 150μl GHLT serum aliquots were inactivated by incubating them at 56°C for 26 minutes, in preparation for the plaque reduction neutralization test (PRNT_90_) [55]. The PRNT_90_ was conducted on Vero cell monolayers (ATCC CCL-81), seeded in six-well plates at a concentration of 1.2 x 10^6^ cells/cm^2^ per well. A standard PRNT_90_ protocol employed at the Laboratory of Arboviruses and Hemorrhagic Viruses at Instituto Oswaldo Cruz, Fiocruz, Rio de Janeiro, Brazil, with a two-day incubation period of the GHLT serum/virus mixture [25,56], was employed. For CHIKV antibody screening, serum samples were initially diluted at 1:10. After screening, reactive sera were again assayed in serial twofold dilutions (1:10 to 1:1280) to titrate neutralizing antibodies. To differentiate between monotypic and heterotypic reactions to CHIKV, the samples were also tested with MAYV, a neotropical alphavirus from the “Semliki Forest” Complex (SFV) [12], both being the most incident alphavirus in Brazil.

An equal to or four-fold difference in neutralizing antibody titers to either of the viruses was considered indicative of the specificity of the reaction. Viral stocks were checked for viral identity to eliminate the possibility of contamination. For confirmation of viral identity, the reference viruses used for PRNT_90_ were subjected to real-time RT-PCR. For CHIKV, a multiplex kit for DENV, Zika (ZIKV), and Chikungunya viruses (Bio-Manguinhos, Brazil) was employed. The real-time RT-PCR for MAYV was conducted as previously described [57]. The viral stocks used in this study were appropriately diluted to a concentration of 80 PFU per well.

Monotypic or heterotypic patterns were distinguished based on whether the serum samples were positive to one or two of the viruses tested. In monotypic situations, samples with a PRNT_90_ titer equal to or greater than 20 for a specific alphavirus and <10 for another alphavirus were considered seropositive. For heterotypic responses, samples exhibiting PRNT_90_ titers for a given alphavirus at least four times higher than those observed for the other tested alphavirus, were considered seropositive. In cases where the difference in titers between any flavivirus was less than four times, samples were considered seropositive for undifferentiated alphaviruses. Samples with titers equal to 10 for a specific *Alphavirus* and <10 for the other alphaviruses were considered inconclusive. Finally, samples with a PRNT_90_ < 10 for all alphaviruses tested *Al* were classified as seronegative [56].

### Data analysis

We calculated the prevalence of neutralizing antibodies for CHIKV in GHLTs, with a 95% of confidence interval (CI). Differences in prevalence among sex, age class, and municipalities were assessed using the Fisher’s Exact test, using a significance level of 95% (p value < 0.05), implemented in R software (version 3.6; Team R Core, 2023).

To compare the scenario of tested animals of Ilhéus and Una with the prevalence of infection, there was analyzed land cover and use of soil (LCSU) patterns, previously defined by MapBiomas organization (by using satellite images), at the areas surrounding the GHLTs. To comprehend and characterize the LCSU we established a 354 m radius buffer around the point of their collection of GHLTs, by using QGIS software (version 3.32; licensed under general public license [40]). This represents 39.4 ha, an average between the 44.7 ha home range of GHLT in the *cabrucas* of Ilhéus [38] and the 34.2 ha in the heterogeneous landscape of Una [58]. The image of LCSU types of the areas was gotten as raster images of the State of Bahia [59] and the MapBiomas Cacau [60] obtained as free available with a resolution of 30 m per pixel. The number of pixels of each kind of LSCU in each buffer was obtained by using the package *raster* of R software, then their percentage was calculated. To compare these data between buffers of each municipality the Shapiro-Wilk test was employed to assess the normality of them, and once they were non-normal, the Mann-Whitney U test was performed, with a significance level of 95% (p value < 0.05).

To understand the proximity between the GHLT home range and human settlements, as the possible source of this urban virus and its vectors, we determined the closest distance between the buffer edges and human settlements on Brazilian Institute of Geography and Statistics (IBGE) census sectors [61], which include urban areas with high building density, urban areas with low building density (encompassing urban expansion areas, new subdivisions, uninhabited green areas, etc.), towns (characterized by the existence of commerce and services), villages (lacking commerce and services like towns), and rural clusters (associated with a single owner, farm, or agricultural establishment) [61]. Files of settlements and limits of political division to perform maps are freely available in Shapefile formats at the webpage of IBGE [62]. Few maps were made in QGIS Open Source software were also performed by using satellite images of bands green, red and infrared (10m resolution) of Sentinel-2 (date: 22/06/09), which are of public domain available from *U*.*S*. *Geological Survey* at Copernicus browser (https://browser.dataspace.copernicus.eu).

## Results

Thirty-two GHLT specimens were captured from the seven different groups located in two designated areas, representing 78,0% of the total population of the known family groups in that area (Table 1) From those groups, 18 specimens were captured in June, July, October, and November from 2021, and 23 individuals were captured in March, April, and May from 2022. Fourteen of these were new captures and nine tamarins were recapture from the first period of 2021, bringing the total to 41 samples (Table 1). The sampling included 40.6% (13/32) females, comprising 12 adults and one juvenile, while males constituted 59.4% (19/32), consisting of 16 adults and three young individuals (one juvenile and two subadults) (Table 1). Throughout the capture period, all the animals exhibited typical social behaviors, maintaining stable group relationships. At the time of sampling, none of the animals displayed clinical signs of disease.

**Table 1:** Distribution of sex and age class, along with CHIKV antibody seropositivity as determined by PRNT_90_, for each free-living group of *Leontopithecus chrysomelas*, Kuhl, 1820 (Golden-Headed Lion Tamarin, GLHT) captured in the municipalities of Ilhéus and Una, south Bahia, Northeastern Brazil, during the period spanning from June to November 2021 and March to May 2022. The group names are abbreviated as follows: Almada (ALM), Bom Pastor (BMP), Santa Rita (SRI), Elias (ELI), Manoel Rosa (MRO), Ozawa (OZW), and Ribeiro (RIB).

		2021						2022						Recaptures in 2022			
Group name	Bahia municipality	Total group size	Sex and age of sampled individuals					Total group size	Sex and age of sampled individuals					Sex and age of sampled individuals			
			AF	JSF	AM (Ab+)	JM	Total		AF	JSF	AM (Ab+)	JM	Total	AF	AM	Total	
ALM	Ilhéus	7	0	0	0	0	0	5	1	0	2 (1)	1	4	0	0	0	
BMP	Ilhéus	7	0	0	2	0	2	8	0	1	2	0	3	0	0	0	
SRI	Ilhéus	4	1	0	2	0	3	4	1	0	0	0	1	0	0	0	
ELI	Una	8	2	0	1	1	4	6	3	0	1	0	4	1	0	1	
MRO	Una	6	1	0	2	0	3	4	1	0	2	0	3	1	2	3	
OZW	Una	8	1	0	2	1	4	9	2	0	3	0	5	1	3	4	
RIB	Una	7	0	0	2 (1)*	0	2	6	2	0	1 (1)*	0	3	0	(1)	(1)	**Total**
		**47**	5	0	11 (1)	2	**18 (1)**	**42**	10	1	11 (2)	1	**23 (2)**	**3**	**6**	**9 (1)**	**41** samples **32** specimens (2)

AF: Adult-Female; JSF: Juvenile-Subadult-Female-; AM: Adult-Male-; JSM: Juvenile-Subadult-Male

(Ab+): Seropositive for CHIKV neutralizing antibodies. * same individual

**Fig 2:**
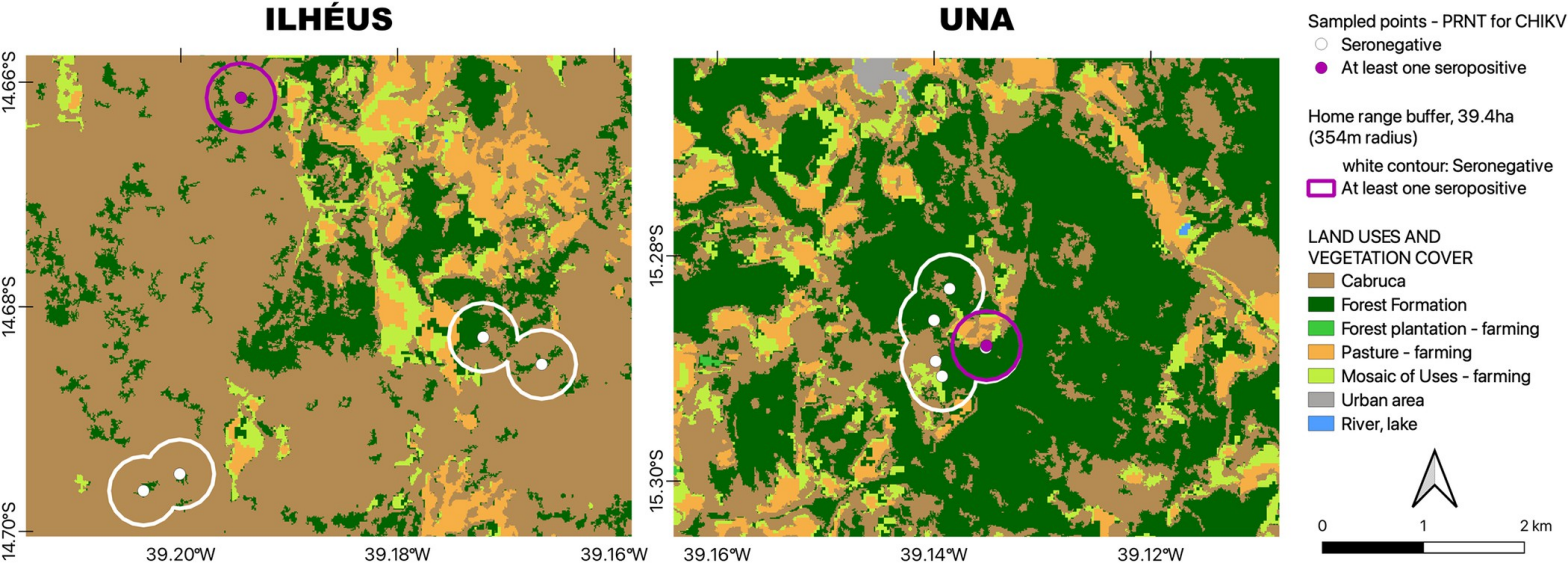
Types of land cover and use of soil based on MapBiomas [59,60] within the study areas in the municipalities of Ilhéus and Una, Bahia, Brazil during the years 2021 and 2022. The depicted areas encompass a 354 m radius home range buffer surrounding the sampling points of *Leontopithecus chrysomelas* (golden-headed lion tamarin, GHLT). MapBiomas Project—Collection 8 of the Annual Series of Land Use Land Cover Maps of Brazil, accessed on 01/02/2024 through the link: https://brasil.mapbiomas.org/colecoes-mapbiomas/ e MapBiomas Cacau–Mapeamento do Cultivo Sombreado de Cacau no Sul da Bahia. accessed on 01/02/2024 through the link: https://brasil.mapbiomas.org/mapbiomas-cacau/. Map generated with the free and open source geographic information system (QGIS,version 3.32; licensed under general public license [40]).

Neutralizing antibodies against CHIKV were detected in 6.3% (2/32; 95% CI: 0–14.6%) of the individuals, displaying a monotypic reaction exclusively for that virus. None of the samples tested positive for MAYV antibodies, excluding cross-reaction with CHIKV. In 2021, was observed a prevalence of 5.6% (1/18; 95% CI: 0–16.1%), and in 2022, a prevalence of 8,7% (2/23; 95% CI: 0–20.2%), with an incidence of 4.5% (1/22, 95% CI: 0–13.2%). This incidence is because the adult male GHLT, #L.24, from the RIB group in Una, whose was recaptured, exhibited detectable neutralizing antibodies in both sampling periords, with titers of 1:20 and 1:10, respectively (Table 2). These antibodies appear to have persisted for at least five months, from November 2021 through April 2022. The other two male adults, #L.33 and #L.31, from the ALM group in Ilhéus, presented titers of 1:20 and 1:10, respectively, in 2022. However, the status of this latter animal was considered inconclusive (Table 2 and Fig 2). Notably, CHIKV RNA was not detected in any of the GHLT blood samples.

**Table 2:** *Leontopithecus chrysomelas*, with neutralizing antibodies anti-CHIKV detected by the PRNT_90_, in non-protected areas from Una and Ilhéus, south Bahia, Brazil in the years 2021 and 2022.

Sample ID	Sex	Stage of development	Group	Bahia Municipality	Sampling day	Antibodies titer	
**L.24**	Male	adult	RIB	Una	12/11/2021	1:20	Seropositive > 5 months
**L.24.2** *					27/04/2022	1:10	
**L.31**	Male	adult	ALM	Ilhéus	27/03/2022	1:10	Inconclusive
**L.34**	Male	adult	ALM	Ilhéus	27/03/2022	1:20	Seropositive

*same animal (captured in 2021 and recaptured in 2022).

While the serological evidence was only identified in two adult males, there was no discernible variation between the sexes and developmental stages among the seropositive GHLTs (Table 3). Although the prevalence for CHIKV infection was higher in Ilhéus than Una, such as 7.7% (95% CI: 0–22.2%) and 5.3% (95% CI: 0–15.3) in Una, respectively, the geographic location did not appear to significantly influence (p value = 1, *Odds ratio*-OR = 1.5) (Table 3).

**Table 3:** Number of animals, prevalence and *odds ratio* (OR) by sex, age and municipality sampling site of neutralizing antibodies against CHIKV in a cohort of 32 free-living *Leontopithecus chrysomelas*, Kuhl, 1820 (Golden-Headed Lion Tamarin, GLHT), from Ilhéus and Una municipalities, south Bahia, Northeastern Region Brazil; during the years 2021 and 2022.

		Seropositive		Seronegative		total	OR (95%CI)	*p value*
Variable		n	%	n	%			
**Sex**	Female	0	0	13	100.0	13	0	0.502
	Male	2	10.5	17	89.5	19		
**Age**	Juvenile / Subadult	0	0.0	4	100.0	4	0	1
	Adult	2	7.1	26	92.9	28		
**Municipality**	Ilhéus	1	7.7	12	92.3	13	1.5	1
	Una*	1	5.3	18	94.7	19		
**Total**		2	6.3	30	93.8	32		

*Group with a recaptured individual in 2021 and 2022.

A total of 128 mosquito specimens were collected, being 25.8% (33/128) in Ilhéus and 74.2% (88/128) in Una. These specimens encompassed eight distinct species from six different genera, being *Tricroposopon digitatum* (28.1%) and *Aedes albopictus* (11.7%) the most abundant. Between all mosquitoes, 88 were tested for CHIKV RNA, yielding negative results (Table 4). The remaining 40 specimens were not tested due to technical constraints.

**Table 4:** Diversity and relative abundance of each species from 88 mosquitoes (Diptera: Culicidae) tested negative for CHIKV RNA, from Ilhéus and Una municipalities, south Bahia, Brazil, in the years 2021 and 2022.

Municipality	Subfamily	Tribe	Taxon	Relative abundance (%)	n tested (not tested)
Ilhéus	Culicinae	Aedini	*Aedes scapularis*	8.6	11 (0)
			*Aedes albopictus*	7.0	7 (2)
			*Aedes (Ochlerotatus)* spp.	0.8	0 (1)
		Mansoniini	*Coquillettidia (Rhyncotaenia)* spp.	1.6	0 (2)
		Sabethinii	*Limatus durhamii*	3.1	4 (0)
			*Wyeomyia (Phoniomyia)* spp.	3.1	0 (4)
			*Wyeomyia (Dendromyia)* spp.	0.8	0 (1)
			*Wyeomyia (Wyeomyia)* spp.	0.8	0 (1)
			**Total**	**25.8**	**22 (11)**
Una	Culicinae	Aedini	*Aedes serratus*	9.4	11 (1)
			*Aedes (Ochlerotatus)* spp.	5.5	5 (2)
			*Aedes albopictus*	4.7	6 (0)
			*Aedes scapularis*	2.3	0 (3)
			*Psorophora ferox*	0.8	0 (1)
		Mansoniini	*Coquillettidia venezuelensis*	3.1	3 (1)
			*Coquillettidia (Rhyncotaenia)* spp.	0.8	0 (1)
		Sabethini	*Trichoprosopon digitatum*	28.1	31 (5)
			*Wyeomyia* spp.	6.2	6 (2)
			*Limatus Durhamii*	3.1	4 (0)
			*Wyeomyia (Phoniomyia)* spp.	3.1	0 (4)
			*Wyeomyia confusa*	2.3	0 (3)
			*Wyeomyia oblita*	1.6	0 (2)
			*Trichoprosopon* spp.	1.6	0 (2)
			*Limatus* spp.	0.8	0 (1)
	Anophelinae	X	*Anopheles (Anopheles) sp*.	0.8	0 (1)
			**Total**	**74.2**	**66 (29)**
			**Total of Ilhéus and Una**	**100**	**88 (40)**

**Fig 3:**
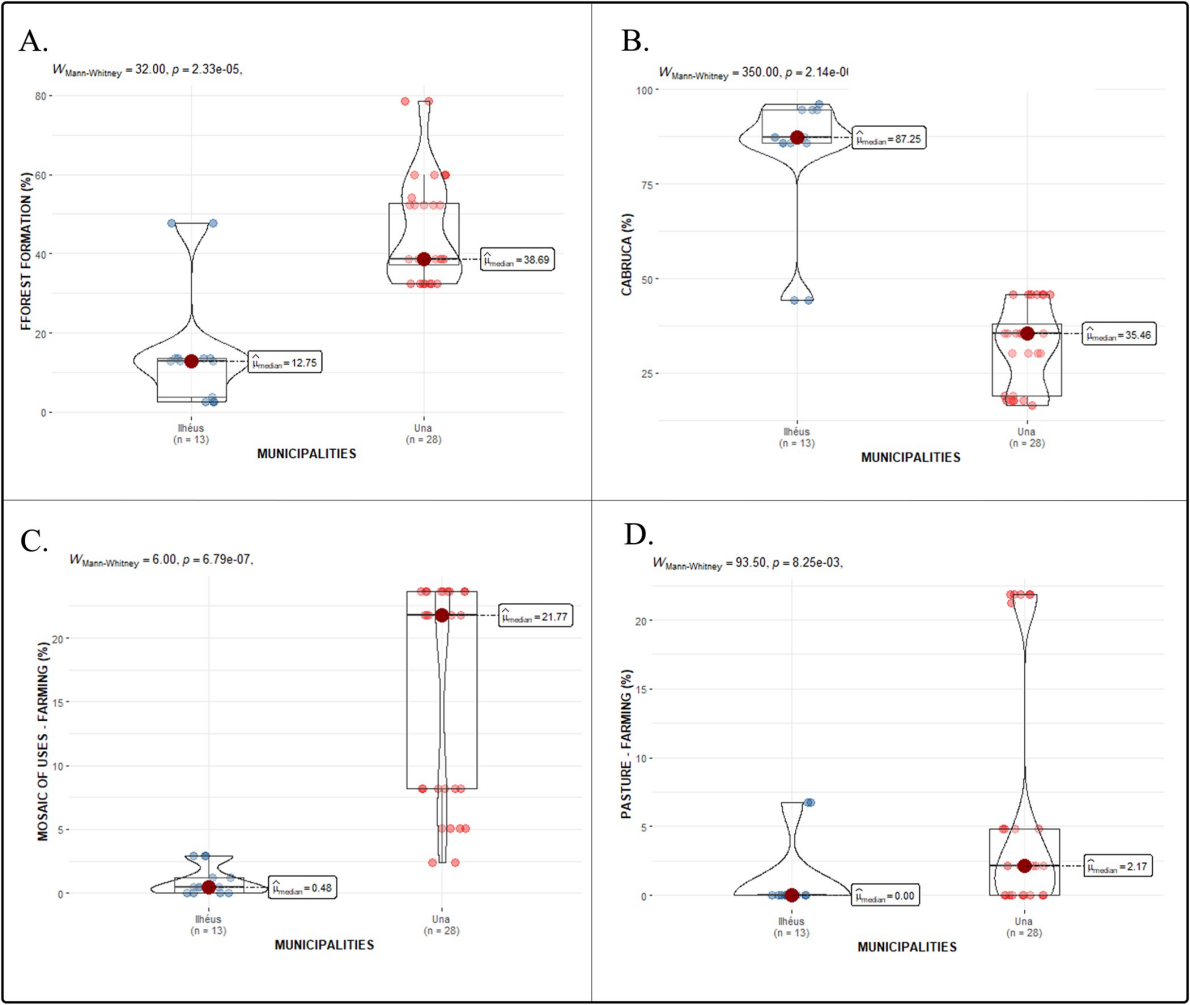
Comparison between median percentages of the different land cover and soil uses (LCSU) types within a 39,4ha (354m radius) home range buffer surrounding the sampling points of *Leontopithecus chrysomelas* (golden-headed lion tamarin, GHLT).

As assumed through the study, the landscape within the various buffers comprised diverse scenarios in Ilhéus and Una, regarding LCSU, such as forest formation, *cabrucas*, mosaic of uses, and pasture, with the latter two primarily dedicated to agricultural and livestock farming purposes (Fig 2). Statistical analysis revealed significant differences in the percentages of all kinds of LCSU between the two municipalities (p<0.05) (Fig 3). Ilhéus was characterized by a predominant presence of *cabrucas*, accounting for 82.60% of the landscape, whereas in Una, *cabrucas* constitute 32.30%. Conversely, Una exhibited a higher prevalence of forest formations, mosaic farming uses, and pasture compared to Ilhéus (Figs 2 and 3).

**Fig 4:**
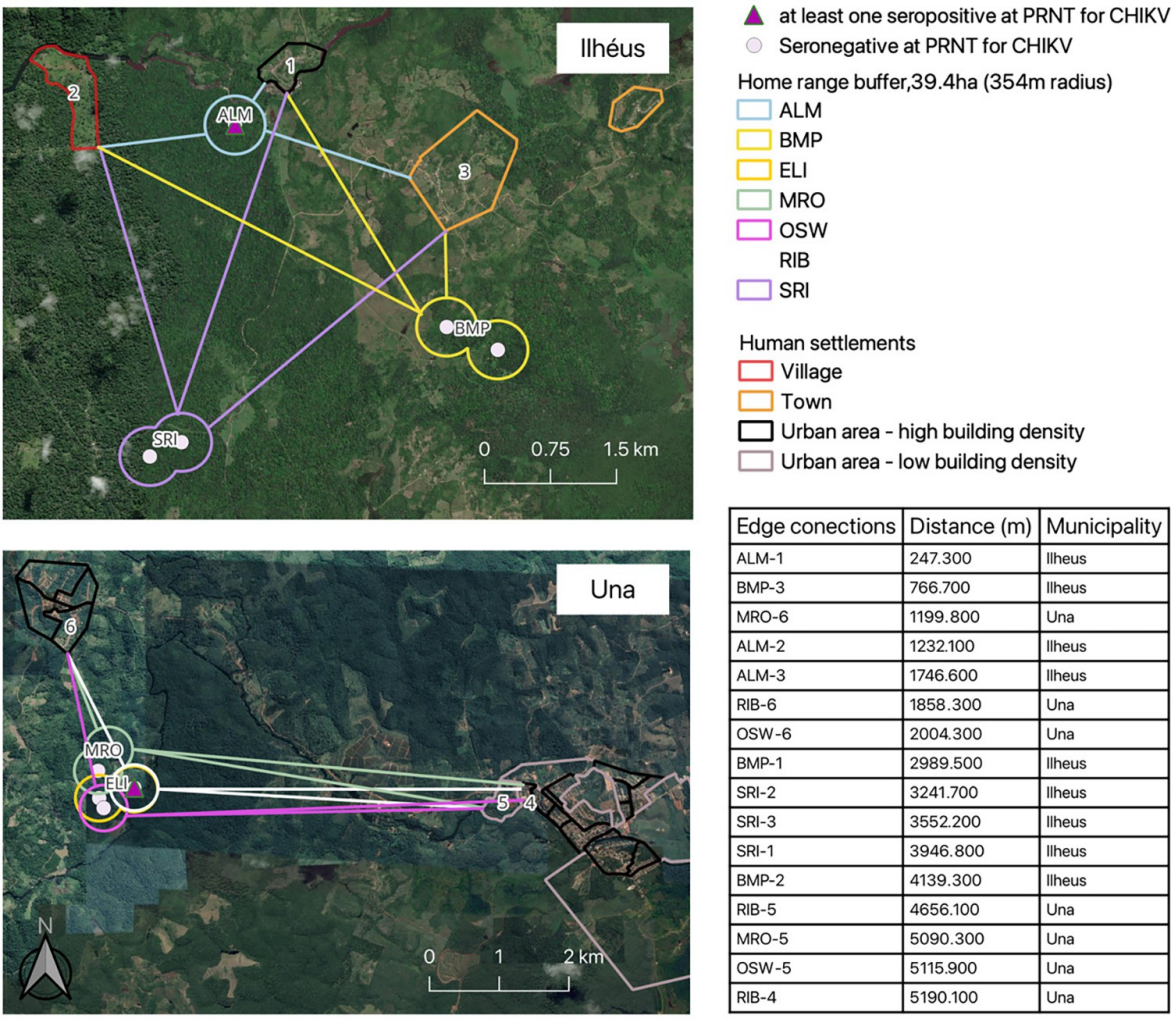
Closest distance edge points of the home range buffer (39.4ha, 354m radius) represented by their group’s names and the human settlements indicated by numbers 1–6, based on IBGE census sectors [61], in the study areas of *Leontopithecus chrysomelas* (golden-headed lion tamarin, GHLT). Group name: Almada (ALM), Bom Pastor (BMP), Santa Rita (SRI), Ribeiro (RIB), Elias (ELI), Manoel Rosa (MRO), and Ozawa (OZW). This image of basemap was performed by using satellite images of bands green, red and infrared (10m resolution) of Sentinel-2 (date: 22/06/09), which are of public domain available from U.S. Geological Survey at Copernicus browser (https://browser.dataspace.copernicus.eu). Map generated with the free and open source geographic information system (QGIS,version 3.32; licensed under general public license [40]).

Regarding the distance between human settlements like town, village and urban area, and the edge of the nearest home range buffer surrounding the GHLT´s capture locations, in Ilhéus the closest was 247.3m, of ALM group and a town (Fig 4, ALM-1) and the farthest was 4,139m, of BMP group from a village (Fig 4, BMP-3). In Una municipality, the closest group from human settlements was ALM, followed by BMP and SRI, such as averages of 1075.3 m, 2631.8 m and 3580.2 m, respectively. Conversely, in Una, the proximity of human settlements varied from as close as 1,199.8 m, of MRO (Fig 4, MRO-6) to as distant as 5,180.6 m, of RIB (Fig 4, RIB-8), relative to the home range edges of the GHLTs (Fig 4 and S2 Table). The farthest group was RIB, followed by MRO and OSW, such as averages of 3901.5 m, 3970.6 m and 4270.7 m, respectively. The closest groups in both municipalities are those with positive samples.

## Discussion

The detection of neutralizing antibodies to CHIKV indicates that *L*. *chrysomelas* situated within or on the periphery of anthropized areas such as *cabrucas*, agroforests, cultivated areas, and secondary forests, as well as in close proximity to human settlements in south Bahia, has been exposed to CHIKV. However, despite this exposure, no CHIKV RNA was detected in GHLTs nor potential mosquito vectors, showing that the infection could be occurred in a previous and indetermined period than the actual research. This study provides the first description of natural exposure to CHIKV in the endangered *L*. *chrysomelas* species.

In the two previous studies where GHLT were tested, no evidence of exposure to CHIKV was detected [25,26]. The first one performed during 2006 and 2014, reported the absence of antibodies to CHIKV or MAYV in 103 GHLTs, seven other NHPs and 29 sloths captured in the same municipalities of Una and Ilhéus, BA [26]. The second study, conducted between 2012 and 2017, did not find neutralizing antibodies against CHIKV in the three GHLTs tested from Bahia and Brasília, but did detected them in eight *Sapajus* spp., one *Ateles marginatus*, and one *Callithrix jacchus* from Bahia, out of a total of 207 tested NHPs. None of those animals were from Ilhéus or Una regions [25]. Thus, our study, along with that of Moreira-Soto and collaborators (2018), indicates a low prevalence of anti-CHIKV antibodies and possible cross-reactivity antibodies with heterologous alphaviruses and flaviviruses [25]. This suggests limited evidence of a relationship between CHIKV and this species of neotropical primates in the region.

Despite the occurrence of human CHIK fever in the State, both before and during the current study, the prevalence in the sampled NHPs remained notably low. Data from 2017 to 2022 indicate a low prevalence of CHIK fever in humans (1.1 per 10,000 inhabitants; 1,607/14,141,626) at the Bahia state. Out of the 1607 confirmed cases of CHIK fever, 104 were in Ilhéus (estimated prevalence of 65,0 per 10,000 inhabitants), and 13 in Una (0.8%; estimated prevalence of 70,1 per 10,000 inhabitants) [26]. Despite the prevalence of Ilhéus and Una being higher than the whole state, they are still low, but can represent the presence of virus at the region.

In urban and peri-urban areas of Northeastern Brazil, where intense local transmission of CHIKV in humans occurs, the frequency of neutralizing antibodies to CHIKV in NHPs was reported to be as low as 5.3% (11/207) [25]. Similarly, GHLT sampled from non-protected forest formations, *cabrucas*, and other farming land uses in rural areas of Ilhéus and Una exhibited a low frequency of 6.3% (2/32), even in the absence of intense local transmission in humans. These observations suggest that despite the limited numbers of specimens sampled in the current study, in conjunction with those of Moreira-Soto et al (2018) [25], the role of these primates in the CHIKV transmission chain seems to be limited. Given that GHLTs are susceptible to virus infection and can maintain neutralizing antibodies for at least five months, as our research showed, or possibly be reinfected during this period, it is plausible that the landscape encompassing rural, peri-rural, and diverse land uses in rural areas allows the natural exposure to the virus. Additionally, it is important to note that antibody duration in other primates [14] and humans [63] can vary, with antibodies potentially lasting for months or even years. This extended duration of antibodies further supports the notion of past exposure without necessarily indicating recent infection or active transmission."

It is crucial to recognize that the detection of antibodies only signifies past exposure, which could have occurred months or even years ago, without providing insights into the viral kinetics or implicating NHPs in the transmission within sylvatic cycles [64]. The earlier observations raise concerns about the potential for spillback from the urban cycle of this arbovirus to a sylvatic cycle, which could prove challenging to manage or eliminate. Fortunately, there is currently no evidence of a wild cycle of CHIKV in Brazil [25].

Viremia in Old World NHPs typically occurs within one to six days post-infection (dpi) with the concomitant production of type I interferon. This narrow timeframe may explain our inability to detect animals with CHIKV RNA. Subsequently, between seven and 35 dpi, CHIKV-specific B and T-cells (CD4+ and CD8+), generate neutralizing antibodies, with titles ranged from 1:20 to 1:640, that remain detectable between 100 and 180 dpi [14]. In humans, PRNT titers can range from 1:10 to 1:2560 or higher, with antibodies remaining detectable for decades after natural infection [63]. Additionally, it appears that CHIKV can establish a persistent infection in NHPs, both Old World or New World species [14].

While our findings demonstrate that GHLTs can maintain neutralizing antibodies for at least five months without showing clinical signs of CHIK fever during the examination periods, it is important to note that the animals were examined at specific intervals, and it is possible that they may have shown clinical signs out of our research time frame. This limitation must be considered when interpreting the results, where broader implications for conservation and survival of the species remain uncertain. The lack of comprehensive understanding of the pathophysiology of CHIKV infection in neotropical NHPs and their role in the virus’s transmission cycle poses challenges in fully assessing potential consequences. Although current evidence suggests limited impact, further research is needed to evaluate the long-term effects on the health and fitness of neotropical NHPs, which could ultimately influence conservation efforts [2,14,25,65].

While CHIKV fever manifestations in humans can range from acute fever and severe chronic arthralgia to neurological signs [12,66,67], the disease in humans pose a low risk of overall mortality. Nevertheless, infection can lead to death, particularly in vulnerable population groups, such as the very young or the elderly [7,66]. More typically, it can lead to significant morbidity, carrying broad socioeconomic impacts and long-term complications [66]. Regarding NHPs, current knowledge is primarily centered around the ones from the Old-World, such as rhesus and cynomolgus macaques, as CHIKV infection in these species mirrors its behavior in humans, characterized by acute fever, rash, joint swelling, and lymphopenia [14]. It is important to highlight that in the clinical examination carried out on the animals in this study, no clinical signs of disease were observed, including those described for Old World NHPs, even in the seropositive animals by the PRNT_90_. However, it is noteworthy that no experimental studies have yet demonstrated CHIKV infection in neotropical NHPs. The study by Lourenço-de-Oliveira and Failloux (2018) indicates the potential for a sylvatic transmission cycle in the Americas, emphasizing the need for further experimental research to understand the role of neotropical NHPs in CHIKV transmission.

In relation to mosquitoes, the majority of those identified in this study belong to widely distributed neotropical species. These mosquitoes are primarily sylvatic but are also prevalent in secondary forests. They have demonstrated adaptability to rural, semi-rural, plantation, and other environments that have been partially modified by human activities [18,19,51,68–71]. The landscape where these mosquitoes thrive is similar to the areas encompassed in this study, inhabited by GHLTs, despite that, we observed more diversity and relative abundance of mosquito species in Ilhéus that has a more diverse landscape and land and soil use than Una, with majority of cabrucas. This uneven distribution highlights the influence of local environmental conditions and anthropic factors on mosquito populations. As reported here, these areas experience high anthropic pressure characterized by deforestation, changes in land use for agriculture, and urbanization, a scenario also documented by Teixeira and collaborators (2023).

The majority of the identified mosquito genera and species detected here have been associated with the transmission of other zoonotic arbovirus or parasites, including CHIKV and YFV [6,16,18,19,49,72,73]. Conversely, we found *Ae*. *albopictus* within the home range of the GHLTs from Una, specifically from OSW group, and small forest fragments near Ilhéus, close to urban areas, but not within the home range of the studied groups, despite a favorable landscape. This Asian invasive species, a major CHIKV vector in some parts of Asia and Europe, is widely distributed in Brazil [74] and is considered a potential “bridge vector” facilitating the transmission between urban and sylvatic cycles [1,6,16,73]. Additionally, *Wy*. *bourrouli* has been found naturally infected with CHIKV in Northeastern Brazil, although its vectorial capacity remains unknown [19]. Active surveillance of mosquito populations, coupled with understanding their current and potential distribution, as well as their ability to transmit various pathogens, is essential. Such knowledge will provide additional tools to support vector control and public health surveillance efforts [19,72].

Although we did not detect CHIKV RNA in any of the mosquitoes examined in the current study, we can presume the potential involvement of *Ae*. *albopictus* in the transmission of CHIKV to the GHLT based on the following considerations: (i) The home range buffers investigated here consisted primarily of anthropically modified lands for agriculture, including *cabrucas* [38], and other cultivated areas [39]. These areas experience daily human movement and are suitable habitats for *Ae*. *albopictus* [75], where humans and wild animals co-exist [38,76]. Notably, despite there being a significant difference in the characteristics of the landscape between the two scenarios (Ilhéus and Una), the prevalence in both did not show different. (ii) The proximity to human settlements, where cases of human CHIKV fever were reported, such as urban areas, villages, and towns in Una and Ilhéus, or even houses within or on the edges of the home range buffers. Some of these locations are within the flight range of female *Ae*. *albopictus*, known to fly up to 1,000 m between sylvatic and human-modified environments [77]. (iii) In addition to ecological and physiological characteristics, blood-feeding preference plays a crucial role in pathogen transmission and control. *Ae*. *albopictus* is traditionally considered a generalist, feeding on both mammals and birds, suggesting that both humans and GHLTs may be sources of their diet. Moreover, in many locations within its native and invasive ranges, *Ae*. *albopictus* has shown a high degree of anthropophily [70,78,79], and in the absence of *Ae*. *aegypti*, this species can be highly productive in urban habitats [70].

The neotropics pose an elevated risk of arbovirus spillover and spillback, particularly at ecotones [80,81], as well as ideal setting for reemergence of urban Yellow Fever Virus [82] or the spillback of CHIKV from the urban cycle into a sylvatic cycle [21,24]. This possibility has been demonstrated by Lourenço-de-Oliveira and Failloux (2017) in Brazil. In our research areas in Una and Ilhéus, all the key components of the arbovirus cycle are present, including the virus, definitive and potential hosts, and potential vectors. Additionally, there are anthropic pressures such as deforestation, changes in land use for agriculture and urbanization [personal observation, [30]]. These environmental factors can contribute to alterations in arboviruses’ ecology and transmission dynamics [50,83,84].

This scenario underscores the critical necessity to enhance environmental, host and vector epidemiological surveys, specially focusing on the vectorial capacity of the local mosquito populations for transmitting arboviruses like CHIKV. In the broader context of epidemiological surveillance for zoonotic arboviruses, understanding how anthropic pressure influences the utilization of habitats by potential sentinel species like NHPs is essential. Moreover, it is crucial to assess the potential consequences for threatened species in the affected ecosystems.

## Conclusion

This study marks the first documented instance of natural exposure to CHIKV in GHLTs, to the best knowledge of the authors. Despite the absence of CHIKV RNA in both NHPs and mosquitoes, including the sampled *Ae*. *albopictus*, a known vector for this virus, the detection of neutralizing antibodies in 6.3% (2/32) of the specimens sampled strongly suggests the occurrence of natural CHIKV exposure in wild *L*. *chrysomelas*. These exposures were observed in GHLTs residing in non-protected forest formations, agroforest fragments like *cabruca*, and various mosaic farming landscapes in South Bahia. Our findings highlight yet another consequence of anthropic pressure on ecosystem health, thus emphasizing the interconnected impact on the health of humans and other animals, aligning with a One health approach. Given the potential of various mosquito species and New World NHPs to harbor and transmit CHIKV, our study highlights the probable existence of a sylvatic cycle involving an endangered species, emphasizing the need for further research, epidemiological surveillance of arboviruses in NHPs and conservation efforts.

## Supporting information

S1 Table Primers and probes used for RT-qPCR assay for the molecular identification of CHIKV [44] in *Leontopithecus chrysomelas*, *Kuhl*, *1820*, from southern Bahia, Brazil. (DOCX)

S2 Table Geographic coordinates of *Leontophitecus chrysomelas* (GHLT) and mosquito capture sites in Una and Ilhéus municipalities, south Bahia, northeastern Brazil; during the years 2021 and 2022. (DOCX)
