# Supplementary material for: Natural exposure to Chikungunya virus in golden-headed lion tamarin (Leontopithecus chrysomelas, Kuhl, 1820) from non-protected areas in southern Bahia, Brazil: Implications and significance
Source: PLoS Negl Trop Dis. 2025 Jan 24;19(1):e0012695. doi: 10.1371/journal.pntd.0012695 (PMC11761120; doi:10.1371/journal.pntd.0012695)
Supplement: S1 Table — (DOCX) [file pntd.0012695.s001.docx]

**Supporting Information**

**S1 Table.** **Primers and probes used for RT-qPCR assay for the molecular identification of CHIKV [44] in *Leontopithecus chrysomelas, Kuhl, 1820,* from southern Bahia, Brazil.**

|  |  | **Primer/Probe mix** | |
| --- | --- | --- | --- |
| **Primer / Probe** | **Sequence (5′o3′)** | **Concentration (μM)** | **Volume (μl)** |
| CHIKV 6856F | TCACTCCCTGTTGGACTTGATAGA | 100 | 50 |
| CHIKV 6981cR | TTGACGAACAGAGTTAGGAACATACC | 100 | 50 |
| CHIKV 6919c_probe | FAM AGGTACGCGCTTCAAGTTCGGCG | 100 | 7,5 |
| Nuclease free H_2_O | - | - | 107,5 |
|  |  |  | 215 |
