# Supplementary material for: Natural exposure to Chikungunya virus in golden-headed lion tamarin (Leontopithecus chrysomelas, Kuhl, 1820) from non-protected areas in southern Bahia, Brazil: Implications and significance
Source: PLoS Negl Trop Dis. 2025 Jan 24;19(1):e0012695. doi: 10.1371/journal.pntd.0012695 (PMC11761120; doi:10.1371/journal.pntd.0012695)
Supplement: S2 Table — (DOCX) [file pntd.0012695.s002.docx]

**S2 Table. Geographic coordinates of *Lentophitecus chrysomelas* (GHLT) and mosquito capture sites in Una and Ilhéus municipalities, south Bahia, northeastern Brazil; during the years 2021 and 2022.**

| **Municipality** | **Collection site ID group** | **Latitude** | **Longitude** | **GHLT** | **Culicidae mosquitoes** |
| --- | --- | --- | --- | --- | --- |
| **Una** | Elias | -15.289362 | -39.139854 | **x** | **x** |
|  |  | -15.288163 | -39.135224 | **x** | **-** |
|  | Ribeiro | -15.28797 | -39.13516 | **x** | **x** |
|  | Manoel Rosa | -15.28572 | -39.14000 | **x** | **-** |
|  |  | -15.28292 | -39.13857 | **x** | **x** |
|  | Ozawa | -15.290690 | -39.139260 | **x** | **x** |
| **Ilhéus** | Almada | -14.661389 | -39.194449 | **x** | **-** |
|  | Bom Pastor | -14.682716 | -39.172095 | **x** | **-** |
|  |  | -14.685130 | -39.166697 | **x** | **-** |
|  | Santa Rita | -14.69489 | -39.20010 | **x** | **-** |
|  |  | -14.69639 | -39.20344 | **x** | **-** |
|  | Center | -14.80595 | -39.02597 | **-** | **x** |
|  |  | -14.81374 | -39.03683 | **-** | **x** |
|  |  | -14.81436 | -39.0383 | **-** | **x** |
|  | North | -14.62423 | -39.07003 | **-** | **x** |
|  |  | -14.62423 | -39.07003 | **-** | **x** |
|  |  | -14.67340 | -39.07484 | **-** | **x** |
|  | South | -14.83966 | -39.0281 | **-** | **x** |
|  |  | -14.83966 | -39.0281 | **-** | **x** |
|  |  | -14.93011 | -39.01935 | **-** | **x** |
